# Yoga Meditation Practitioners Exhibit Greater Gray Matter Volume and Fewer Reported Cognitive Failures: Results of a Preliminary Voxel-Based Morphometric Analysis

**DOI:** 10.1155/2012/821307

**Published:** 2012-12-05

**Authors:** Brett Froeliger, Eric L. Garland, F. Joseph McClernon

**Affiliations:** ^1^Department of Psychiatry and Behavioral Sciences, Duke University Medical Center, Durham, NC 27708, USA; ^2^Brain Imaging and Analysis Center, Duke University Medical Center, Durham, NC 27708, USA; ^3^College of Social Work, Florida State University, Tallahassee, FL, USA; ^4^Trinity Institute for the Addictions, Florida State University, Tallahassee, FL, USA

## Abstract

Hatha yoga techniques, including physical postures (asanas), breathing exercises (pranayama), and meditation, involve the practice of mindfulness. In turn, yoga meditation practices may induce the state of mindfulness, which, when evoked recurrently through repeated practice, may accrue into trait or dispositional mindfulness. Putatively, these changes may be mediated by experience-dependent neuroplastic changes. Though prior studies have identified differences in gray matter volume (GMV) between long-term mindfulness practitioners and controls, no studies to date have reported on whether yoga meditation is associated with GMV differences. The present study investigated GMV differences between yoga meditation practitioners (YMP) and a matched control group (CG). The YMP group exhibited greater GM volume in frontal, limbic, temporal, occipital, and cerebellar regions; whereas the CG had no greater regional greater GMV. In addition, the YMP group reported significantly fewer cognitive failures on the Cognitive Failures Questionnaire (CFQ), the magnitude of which was positively correlated with GMV in numerous regions identified in the primary analysis. Lastly, GMV was positively correlated with the duration of yoga practice. Results from this preliminary study suggest that hatha yoga practice may be associated with the promotion of neuroplastic changes in executive brain systems, which may confer therapeutic benefits that accrue with repeated practice.

## 1. Introduction

Hatha yoga techniques, including physical postures (asanas), breathing exercises (pranayama), and meditation, involve the practice of mindfulness, that is, repeated placement of attention onto an object while alternately acknowledging and letting go of distracting thoughts and emotions. In the case of yoga, the object of mindfulness practice might include proprioceptive or interoceptive sensations stemming from physical posture or respiration. In turn, mindful yoga practices may induce the state of mindfulness, which, when evoked recurrently through repeated practice, may accrue into trait or dispositional mindfulness [1, 2]. The state of mindfulness is characterized by a nonjudgmental and metacognitive monitoring of momentary thoughts, emotions, action urges, perceptions, and bodily sensations [3–5]. Correspondingly, trait mindfulness is characterized as the propensity to adopt a nonjudgmental awareness of these present-moment experiences in everyday life [6]. Trait mindfulness can be promoted by repeated practice of mindfulness meditation [7, 8]. However, the practice of yoga may also engender mindfulness: in that regard, participants in a yoga intervention exhibited significant increases in trait mindfulness after eight weeks of training [9]. Another study demonstrated that participation in a residential yoga intervention was associated with increases in mindfulness that mediated the effects of yoga on quality of life [10]. In addition, prospective observational research indicates that the trait mindfulness increases and is maintained over the course of six months of yoga training [11]. Thus, research suggests that individuals may develop greater mindful awareness over time as a result of disciplined practice of yoga and meditation. 

A number of studies link mindfulness with enhanced cognitive function and brain plasticity (for reviews see [12, 13]). For example, mindfulness practice has been shown to promote attentional regulation [5, 14–16] and increased executive control of automatic responses [17–20]. When sustained over longer periods of time, the practice of mindfulness through meditation and yoga may promote durable, trait-like alterations in domain-general forms of cognitive control. In other words, pursuit of these contemplative practices should confer general functional enhancement beyond an improved ability to implement yoga and meditation techniques; such process-specific learning should generalize to a wider range of skills needed for effective performance in everyday life [13]. Concomitantly, positive associations have been observed between trait mindfulness and cognitive control [21, 22], whereas negative associations have been found between trait mindfulness and mind wandering [23]. Theorists suggest that lasting functional improvements may derive from mindfulness-induced neuroplasticity in brain regions that instantiate basic cognitive processes [13, 24].

Thus, practices designed to cultivate mindfulness, such as yoga, may operate through a fundamental *state-by-trait interaction*, such that repeated activation of the mindful state (and the neural networks that instantiate this state) via contemplative practices (e.g., *asanas*, *pranayama*, or meditation) may leave lasting psychobiological traces that accrue into durable changes in trait mindfulness and cognitive function [2, 14]. Putatively, these changes may be mediated by experience-dependent alterations in gene expression resulting in neuroplasticity [24–26]. In support of this hypothesis, a number of studies using voxel-based morphometry have identified significant differences in gray matter concentration between long-term mindfulness practitioners and controls [27–29]. Moreover, recent longitudinal research suggests that participating in 8 weeks of mindfulness meditation training is associated with increases in gray matter density in the left hippocampus, posterior cingulate cortex, and temporo-parietal junction, brain structures believed to be central to cognitive processes such as memory and attention [30]. Theoretically, such neuroplastic changes in brain structure arise from the recurrent activation of corresponding functional networks during repeated practice of mindfulness. Should yoga and meditation prove to be a reliable means of inducing brain plasticity, these contemplative practices may hold promise as means of neurocognitive rehabilitation for clinical populations suffering from cognitive failures like lapses in attention, absent-mindedness, and other executive function impairments.

The purpose of the present study was to explore differences in brain structure and neurocognitive function between practitioners of hatha yoga meditation and a sample of meditation-naïve controls (CG). We hypothesized that relative to the CG, the hatha yoga meditation practitioners (YMP) would exhibit significantly greater gray matter concentration in prefrontal cortex and hippocampus and significantly less self-reported cognitive failures. Furthermore, we hypothesized that these structural differences would be associated with fewer self-reported cognitive failures. 

## 2. Methods

### 2.1. Participants

Fourteen (7 hatha yoga meditation practitioner (YMP), 7 hatha yoga and meditation-naïve control (CG)) participants between the ages of 18 and 55 years were enrolled. YMP participants reported maintaining an active and ongoing modern hatha yoga practice (>45-minutes per day, three-four times per week, >three years (*M* = 9.4; SD = 2.4)) and engaging in mindfulness meditation on average 7 days per week (0) over the course of the previous 5.6 yrs (4.2). The matched control group reported no current or past dedicated meditation or yoga practice. In addition, all participants were right-handed, free of any psychiatric condition or any major medical condition that would make participation unsafe or uncomfortable. Additional exclusionary criteria included current alcohol or drug abuse, use of tobacco or nicotine products, and positive urine drug screen. Female participants were required to have a negative urine pregnancy test at screening and within 12 hours prior to the MRI scan. The protocol was approved by the institutional review board at Duke University Medical Center, and all participants provided written informed consent before participating in study-related activities.

### 2.2. Assessment of Baseline Mood and Cognition

Baseline measures included assessment of depressive symptoms with the Center for Epidemiological Studies-Depression (CES-D) scale [31] and anxiety symptoms with the Beck Anxiety Inventory (BAI). State-dependent mood was measured using the 20-item positive and negative affect schedule (PANAS) [32]. This measure results in two orthogonal scales—positive affect (attentive, proud) and negative affect (distressed, angry). Cognitive failures were assessed with the Cognitive Failures Questionnaire (CFQ) [33]. This measure assesses the frequency with which participants experience lapses in executive function, including attention, memory, and motor coordination, as exemplified by items such as “Do you find you forget why you went from one part of the house to the other?”, “Do you bump into people?”, “Do you have trouble making up your mind?”, “Do you daydream when you ought to be listening to something?”, “Do you start doing one thing at home and get distracted into doing something else?”, and “Do you drop things?”

### 2.3. Procedures

Eligible participants underwent one MRI session where anatomical scans were acquired as part of a larger functional neuroimaging protocol [34, 35].

#### 2.3.1. MRI Methods

**(1) Image Parameters**

A 3 T GE Discovery MR750 scanner with 41 mT/m gradients was used for image acquisition. Each participant's head was held in place using a vacuum-pack system to minimize head motion. A high-resolution spoiled gradient recalled (FSPGR) anatomical image (124 slices, 1 mm thick) was acquired with *T*_*R*_ = 7.58 ms, *T*_*E*_ = 2.9 ms, FOV = 256 × 256, and in-plane resolution = 1 mm^3^.

**(2) Voxel-Based Morphometry**

Voxel-based morphometry (VBM) with DARTEL [36] was conducted using SPM8 (Wellcome) implemented in Matlab 7.0.4. VBM with DARTEL has been shown to be more sensitive than standard VBM [37] and provides results comparable to those achieved with manual segmentation [38]. Each participant's anatomical image was manually aligned to the MNI template and then segmented into gray matter (GM), white matter (WM), and cerebrospinal fluid (CSF) images. Using the DARTEL algorithm, nonlinear deformations that best aligned each participant's image to a study-specific template were estimated. The registered images were then multiplied with the Jacobian determinants of the deformations in order to preserve relative tissue volumes in each structure. Each modulated, warped GM image was then transformed to MNI space [38]. GM images were then smoothed using an 8 mm FWHM Gaussian filter. Finally, a binary mask that eliminated any voxels with an absolute GM threshold less than 0.05 was applied [39–41].

### 2.4. Statistical Analysis

The primary goal of examining differences in GM volume between groups was achieved by conducting a two-sample *t*-test in SPM. In order to control inherent differences in brain structures, total intracranial volume (TIV), age, sex, and education were included as nuisance covariates. TIV was calculated as the sum of segmented images of modulated gray matter, white matter, and CSF [38]. Clusters in this analysis were considered significant at *P* < 0.05 cluster corrected (3008 *μ*L cluster of contiguous voxels at *P* < 0.005) as determined through 1,000 Monte Carlo simulations [42].

Furthermore, exploratory regression analyses were performed to examine relations between (1) self-reported cognitive failures; (2) yoga experience and GM volume within a functional gray matter mask generated from the significant clusters identified in the primary analysis of group differences. 

To explore correlations between self-reported cognitive failures and GM volume, the composite score from the CFQ for each subject was entered a regressor of interest. To explore correlations between yoga experience and GM volume, years of yoga practice for each subject were entered a regressor of interest. In each model TIV, age, sex, and education were included as nuisance covariates. Activation in the regression analyses were considered significant at *P* < 0.05 cluster corrected (69 *μ*L cluster of contiguous voxels at *P* < 0.005) as determined through 1,000 Monte Carlo simulations [42].

## 3. Results

### 3.1. Participant Demographics and Self-Report

Detailed results for group demographics and self-report data may be found in Table 1. In brief, no between-group differences were found on demographic variables, or baseline depression, anxiety, or mood. A significant main effect of group was found for the total score on the Cognitive Failures Questionnaire (CFQ), *t (2-tailed)* = 8.1, *P* < 0.000; the YMP group reported a lower mean score (*M* = 33.1(5.7)) as compared to the control group (*M* = 75.4(12.6)). 

### 3.2. VBM Results

#### 3.2.1. Group Differences

**Figure 1:**
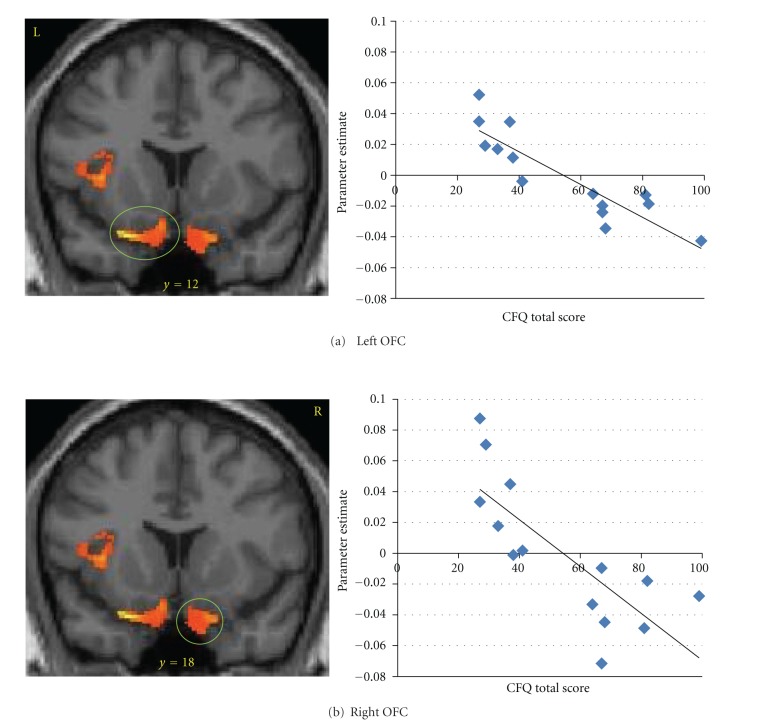
Gray matter volume (GMV) was greater in yoga mediation practitioners as compared to controls in (a) left orbital frontal cortex (OFC; −22, 12, −21) and (b) right OFC (6, 18, −19). OFC GMV was negatively correlated with self-reported cognitive failures.

Detailed results from the VBM analysis of the main effect of group differences can be found in Table 2. After controlling all covariates, compared to controls, YMP exhibited significantly higher GM volume in a number of regions including frontal (i.e., bilateral orbital frontal, right middle frontal, and left precentral gyri) (see Figure 1), limbic (i.e., left parahippocampal gyrus, hippocampus, and insula), temporal (i.e., left superior temporal gyrus), occipital (i.e., right lingual gyrus) lobes and cerebellum. No regions were identified where controls had greater GM volume that YMP.

#### 3.2.2. Cognitive Failures and Gray Matter Volume

Detailed results from the regression analyses exploring correlations between self-reported cognitive failures and GM volume can be found in Table 3. Within the GM volume mask of clusters where significant group differences were identified, the total score on the CFQ was negatively correlated with GM volume in frontal (see Figure 1), limbic temporal, occipital, and cerebellar regions. No positive correlations between CFQ total score and GM volume were found.

#### 3.2.3. Yoga Experience and Gray Matter Volume

Detailed results from the regression analyses exploring correlations between yoga experience and GM volume can be found in Table 4. Within the GM volume mask of clusters where significant group differences were identified, years of yoga practice were positively correlated with GM volume in frontal, limbic, temporal, occipital, and cerebellar regions. No negative correlations between years of yoga practice and GM volume were found.

## 4. Discussion

The present study identified significant differences in gray matter volume and self-reported cognitive failures between hatha yoga meditation practitioners (YMP) and a sample of well-matched controls (CG), such that YMP exhibited volumetrically larger brain structures and fewer lapses in executive function in daily life. Structural differences were particularly evident in brain regions subserving higher-order control of cognitive and motor responses. Concomitantly, the extent to which YMP and CG differed with regard to gray matter volume in these regions was significantly associated with the occurrence of self-reported cognitive failures. Moreover, yoga meditation experience was significantly predictive of gray matter volume in many of these same neuroanatomical regions. Taken together, study findings suggest that the practice of hatha yoga (a multimodal discipline involving physical postures, breathing exercises, and meditation) is associated with enhanced cognitive function coupled with enlargement of brain structures held to instantiate executive control.

 VBM analysis indicated that, on the whole, YMP exhibited significantly larger prefrontal cortical regions (including the middle and orbital frontal gyri) than the CG. Experimental and legion studies indicate these brain structures are recruited during tasks that involve cognitive control [43], inhibition of automatized or prepotent responses [44], the contextually appropriate selection and coordination of actions [45], and reward evaluation and decision making [46, 47]. Self-report data from the Cognitive Failures Questionnaire [33] indicate that greater gray matter volume in these regions was associated with making fewer errors in attention, memory, and motor function in everyday tasks. Relative to the CG, YMP also exhibited significantly greater gray matter volume in the cerebellum, a brain structure known for decades as integral to the precise coordination and timing of body movements [48], but more recently has been acknowledged to be involved in executive function [49]. Common to both of these domains, the cerebellum may predict the consequences of planned actions, be they motor behaviors or mental operations, and use these predictions to update action plans [50]. Putatively, the integration of cognitive and motor control is mediated by anatomical connections between units in the cerebellum and regions of prefrontal cortex [51]. 

Hypothetically, prolonged practice of hatha yoga might stimulate frontocerebellar connectivity and neuroplasticity by virtue of the intense, multimodal, cognitive, and motor skill learning that such practice involves. The word yoga, stemming from the Sanskrit word *yuj*, “to yoke” or “bind together,” refers to the primary aim of the practice: to unify mind and body by cultivating heightened mindfulness and self-discipline, ultimately leading to equanimity and insight [52]. Hatha yoga involves the complex training context, high task variability, increasing task difficulty, motivated states of arousal, and long duration of training believed to be requisite to process-specific learning undergirded by brain plasticity [13]. Indeed, the practice of hatha yoga demands exquisite executive control to coordinate body posture and breathing while maintaining attentional focus on proprioceptive and interoceptive feedback in the face of distracting thoughts and bodily discomfort. In addition, ardent motivation is needed to endure the rigors of yoga, which increase in difficulty as the practice deepens. Great precision is required to move into progressively more challenging physical postures while timing the positioning of limbs with respiration. In light of these characteristics, disciplined pursuit of yoga meditation may foster cognitive plasticity through the intensive mental training this practice entails.

Although study findings are preliminary, they suggest that yoga and/or meditation practice may serve as an effective treatment intervention for disorders with concomitant GM volume atrophy and cognitive difficulties. For example, results from the current study may be meaningfully contrasted with extant literature demonstrating that GM atrophy is associated with a broad array of psychiatric conditions including depression [53], age-related mild cognitive impairment and depression [54], posttraumatic stress disorder [55, 56], and chronic pain [57]. In addition, substance use disorders are associated with decreased GM volume; including addictive use of alcohol [58], cigarettes [59, 60], and psychostimulants [61]. Importantly, GM volume reductions in frontal and limbic regions are found to be associated with deficits in cognition function [54, 61]. Consistent with the hypothesis that yoga meditation practice may remediate psychiatric conditions, a recent review paper of over 90 studies found that mind-body therapies improved depressive symptoms in patients suffering from a wide range of ailments [62]. With regard to addictive disorders, Yoga has been reported to improve recovery from substance abuse disorders [63] and improve smoking cessation outcomes among nicotine dependent individuals [64]. Whether such therapeutic benefits derive from increased mindfulness and neuroplasticity stemming from state-by-trait interactions remains to be determined by future research.

To be clear, due to the cross-sectional nature of this study, no causal inferences can be drawn between the practice of hatha yoga, increased gray matter volume, and cognitive function. Indeed, it is possible that the observed neuroanatomical and cognitive differences between YMP and CG were extant prior to the initiation of hatha yoga and may reflect a preexisting propensity to engage in contemplative practice. However, the fact that number of years of yoga experience was significantly associated with gray matter volume suggests that duration of yoga practice may contribute in part to the observed volumetric differences in brain structure, possibly by stimulating neuroplasticity. In addition, the current study design did not allow us to differentiate effects that may be due to yoga versus those that may be due to meditation outside of the context of any yoga practice. Future research may address this issue by comparing yoga practitioners with yoga-naïve meditation practitioners. Furthermore, the modest sample size may have limited the statistical power of our analyses. In this regard, it is notable that robust between-groups differences were observed for gray matter volume and self-reported cognitive failures. Future research should readress these limitations by employing randomized, controlled, longitudinal designs, where yoga-naïve subjects are scanned at baseline, randomly allocated to receive either yoga training or a comparable control group and then followed for a prolonged period of time before receiving additional MRI. In addition, behavioral measures of executive function (e.g., GoNoGo task, Stop Signal Task) should be used to assess whether volumetric differences between YMP and CG correspond with objective indices of cognitive performance enhancement.

## Figures and Tables

**Table 1:** Subject demographics and self-report.

	YMP (*n* = 7)	Controls (*n* = 7)	Group differences
No. Female	6	6	
Mean age (SD)	36.4 (11.9)	35.5 (7.1)	
Years of education (SD)	15.5 (2.5)	15.3 (2.3)	
Years of yoga (SD)	9.3 (2.4)	0	
Years of meditation (SD)	5.6 (4.2)	0	

Baseline mood and cognition			

BAI	14.4 (2.5)	12.5 (1.9)	*ns* [>0.15]
CESD	3.4 (3.8)	2.6 (3.2)	*ns* [>0.6]
PANAS: positive	35.6 (9.0)	36.1 (10.3)	*ns* [>0.9]
PANAS: negative	10.4 (0.8)	10.7 (1.9)	*ns* [>0.7]
Cognitive failures (CFQ)	33.1 (5.7)	75.4 (12.6)	*t* = 8.1, *P* < 0.000

**Table 2:** VBM analyses of great matter volume differences between yoga meditation practitioners and controls.

Yogis > controls					TAL		Cluster size (mm^3^)	*Z* (max)
Hemisphere	Lobe	Brain region	Brodmann area	*X*	*Y*	*Z*		
R	Frontal	Orbital frontal gyrus	25/47	6	18	−19	3176	4.37
L	Frontal	Orbital frontal gyrus	11/47	−22	12	−21	4280	4.14
L	Frontal	Precentral/middle frontal gyrus	4/6	−45	−11	50	5448	4.22
L	Limbic	Parahippocampal gyrus/hippocampus	36	−34	−30	−16	4008	3.65
L	Limbic	Insula		−34	15	3	3096	3.43
L	Temporal	Superior temporal gyrus	38	−48	−6	−10	5016	4.50
R	Occipital	Lingual gyrus	18/19	12	−82	−7	10968	4.56
L	Cerebellum	Posterior		−1	−64	−28	3568	4.54
L	Cerebellum	Anterior		−30	−38	−26	4544	3.59

Controls > yogis								

		None						

**Table 3:** Regions within VBM group differences maskwherecognitive failures is negatively correlated with gray matter volume.

Hemisphere	Lobe	Brain region	Brodmann area	MNI			Cluster size (mm^3^)	*Z* (max)	*R* ^2^
				*X*	*Y*	*Z*			
R	Frontal	Orbital frontal gyrus	47	20	15	−26	192	2.88	0.61
L	Frontal	Orbital frontal gyrus	47	−27	15	−23	912	3.86	0.78
L	Parietal	Postcentral/precentral/middle frontal gyrus	3/4/6	−51	−16	52	1952	4.3	0.83
L	Frontal	Precentral gyrus	44	−44	12	6	280	2.93	0.62
L	Temporal	Superior temporal gyrus	38	−50	9	−15	2216	4.73	0.86
L	Limbic	Insula	13	−36	9	3	344	2.98	0.63
R	Limbic	Uncus	20	33	−16	−24	352	2.93	0.62
R	Occipital	Lingual gyrus	18	20	−78	−8	7944	4.73	0.86
L	Cerebellum	Inferior semilunar lobule		−5	−70	−39	2560	3.85	0.78
L	Cerebellum	Tuber		−42	−58	−29	1816	3.54	0.86
L	Cerebellum	Inferior semi-lunar lobule		−39	−69	−44	776	3.11	0.67

**Table 4:** Regions within VBM group differences maskwhereYMP experience (years) is positively correlated with gray matter volume.

Hemisphere	Lobe	Brain region	Brodmann area	MNI			Cluster size (mm^3^)	Z (max)	*R* ^2^
				*X*	*Y*	*Z*			
R	Frontal	Rectal gyrus	11	9	21	−23	1688	3.86	0.78
L	Frontal	Precentral/middle frontal gyrus	4/6	−44	−10	54	2976	4.18	0.82
L	Frontal	Orbital frontal gyrus	47	−23	20	−24	2520	4.05	0.81
L	Limbic	Parahippocampal gyrus	35	−23	−21	−20	200	3.54	0.74
L	Limbic	Fusiform/parahippocampal gyrus	20/36	−36	−28	−21	352	3.14	0.66
L	Temporal	Superior temporal gyrus	21/38	−50	−7	−12	3088	4.68	0.86
R	Occipital	Lingual gyrus	18	14	−84	−14	5352	3.78	0.77
L	Cerebellum	Inferior semilunar lobule		−3	−61	−39	2008	3.86	0.78
L	Cerebellum	Cerebellar tonsil		−30	−37	−33	536	2.92	0.62
